# Store-operated calcium entry is essential for glial calcium signalling in CNS white matter

**DOI:** 10.1007/s00429-017-1380-8

**Published:** 2017-02-28

**Authors:** M. Papanikolaou, A. Lewis, A. M. Butt

**Affiliations:** Institute of Biomedical and Biomolecular Sciences, School of Pharmacy and Biomedical Science, University of Portsmouth, Portsmouth, PO1 2DT UK

**Keywords:** Store-operated calcium channel, CRAC, TRP channel, Glia, Astrocyte, Oligodendrocyte, White matter, Calcium signalling

## Abstract

‘Calcium signalling’ is the ubiquitous response of glial cells to multiple extracellular stimuli. The primary mechanism of glial calcium signalling is by release of calcium from intracellular stores of the endoplasmic reticulum (ER). Replenishment of ER Ca^2+^ stores relies on store-operated calcium entry (SOCE). However, despite the importance of calcium signalling in glial cells, little is known about their mechanisms of SOCE. Here, we investigated SOCE in glia of the mouse optic nerve, a typical CNS white matter tract that comprises bundles of myelinated axons and the oligodendrocytes and astrocytes that support them. Using quantitative RT-PCR, we identified Orai1 channels, both Stim1 and Stim2, and the transient receptor potential M3 channel (TRPM3) as the primary channels for SOCE in the optic nerve, and their expression in both astrocytes and oligodendrocytes was demonstrated by immunolabelling of optic nerve sections and cultures. The functional importance of SOCE was demonstrated by fluo-4 calcium imaging on isolated intact optic nerves and optic nerve cultures. Removal of extracellular calcium ([Ca^2+^]_o_) resulted in a marked depletion of glial cytosolic calcium ([Ca^2+^]_i_), which recovered rapidly on restoration of [Ca^2+^]_o_ via SOCE. 2-aminoethoxydiphenylborane (2APB) significantly decreased SOCE and severely attenuated ATP-mediated calcium signalling. The results provide evidence that Orai/Stim and TRPM3 are important components of the ‘calcium toolkit’ that underpins SOCE and the sustainability of calcium signalling in white matter glia.

## Introduction

Glial cells respond to a wide range of extracellular stimuli, including neurotransmitters, by a rise in cytosolic calcium ([Ca^2+^]_i_) termed ‘Ca^2+^ signalling’, which is the basis of ‘glial excitability’ (Khakh and McCarthy 2015). Glial calcium signalling regulates the release of ‘gliotransmitters’, such as ATP, which couple glia within a panglial syncytium and act on neurones and cerebral vasculature to modulate neuronal activity and cerebral blood flow, with important roles in regulating glial physiological functions and pathology (Butt et al. 2014; Fern et al. 2014). Glial calcium signalling is mainly dependent on inositol 1,4,5-triphosphate (IP3)-mediated release of Ca^2+^ from the endoplasmic reticulum (ER), which depends on the replenishment of intracellular stores through store-operated calcium entry (SOCE) (Verkhratsky and Parpura 2014). The two main plasmalemmal channels mediating SOCE are transient receptor potential (TRP) channels, comprising six subfamilies, and calcium release-activated calcium channels (CRAC), which are formed of Orai, the plasmalemma spanning channels and STIM (Stromal Interaction Molecule), which function as ER Ca^2+^ sensors (Verkhratsky and Parpura 2014). Depletion of ER Ca^2+^ triggers STIM to interact with plasmalemmal SOCE channels, both Orai and TRP (Huang et al. 2006; Mercer et al. 2006), resulting in influx of Ca^2+^ from the extracellular milieu into the cytosol and subsequent refilling of ER stores through uptake via calcium (SERCA) pumps on the ER membrane (Jousset et al. 2007). SOCE has been reported in astrocytes (Golovina 2005; Pivneva et al. 2008) and oligodendrocytes (Hoffmann et al. 2010) and, although the molecular mechanisms have not been fully resolved, it has been generally considered that astrocytes and oligodendrocytes depend mainly on TRP channels, whilst Orai are the predominant mechanism in microglia (Verkhratsky and Parpura 2014). In this study, we show that astrocytes and oligodendrocytes express both Orai/Stim and TRP channels in the mouse optic nerve, a typical CNS white matter tract.

White matter comprises bundles of myelinated axons and the glia that support them, ensuring the rapid communication between the different parts of the CNS that underlies the massive computing power of the brain (Butt et al. 2014). Hence, disruption of white matter, such as occurs in the demyelinating disease multiple sclerosis (MS), has devastating effect on CNS function (Fern et al. 2014). Despite the lack of neuronal cell bodies and synapses in white matter, neurotransmitter signalling is prominent and mediates axon–glial communication, which evokes Ca^2+^ signalling in astrocytes and oligodendrocytes and serves to couple their homeostatic and myelinating functions to axonal activity, thereby ensuring rapid electrical conduction (Butt et al. 2014). A key feature in CNS white matter glia is the predominance of ATP-mediated IP3-dependent Ca^2+^ signalling, which has a clearly established role in glial physiology and pathology, including MS, stroke and traumatic injury (Rivera et al. 2016; Langer et al. 2017). However, despite the prominence of Ca^2+^ signalling in CNS white matter, the molecular mechanisms of SOCE have not been fully resolved. Here, we show that the SOCE channels TRPM3 and Orai/Stim are expressed by astrocytes and oligodendrocytes in the mouse optic nerve, and SOCE is shown to be essential for glial Ca^2+^ calcium signalling in this typical white matter tract.

## Materials and methods

### Experimental animals

All animals were killed humanely by cervical dislocation, in accordance with regulations issued by the Home Office of the UK under the Animals (Scientific Procedures) Act, 1986. The animals used were C57BL6/10 wild type (WT) or PLP-DsRed transgenic mice, in which DsRed is under the control of the oligodendrocyte-specific proteolipid protein (PLP) promoter (Hirrlinger et al. 2005), kindly provided by Frank Kirchhoff (Molecular Physiology, University of Saarland, Homburg, Germany). Optic nerves were dissected free and placed immediately in either: (1) Buffer RLT (Qiagen) for RNA extraction and qRT-PCR; (2) dissecting medium for explant cultures; (3) 41% paraformaldehyde (PFA) for immunohistochemistry; or (4) artificial cerebrospinal fluid (*a*CSF) for calcium imaging (details below).

### qRT-PCR

Maintaining strict RNase-free and sterile conditions throughout, RNA extraction was performed using published protocols (Azim et al. 2014). RNA was processed using an RNeasy Micro kit (Qiagen) and converted to single-stranded cDNA using the RT^2^ First Strand Kit (Qiagen), following manufacturer’s instructions. The quantity of RNA that was transcribed was the same for all samples (500 ng). cDNA libraries were prepared from total RNA extracted from 10 pooled optic nerves from WT postnatal mice [aged postnatal day (P)9–12], and adult mice (aged P30–40), and analyses were run in triplicate. SYBR Green qPCR Mastermix (Qiagen) was mixed with cDNA and ultra-pure water (Ambion) and 25 μl was pipetted in each well of the 96 well-plate arrays for the Lightcycler 96 (Roche), using the Mouse Neuronal Ion Channels RT^2^ Profiler™ qPCR array and a custom RT² Profiler™ qPCR array for additional channels not included in the neuronal array, namely STIM1, STIM2, IP3R1, IP3R2, TRPC2, TRPM3, TRPM4, TRPM7 and TRPV1 (Sabiosciences, Qiagen). Relative gene expression was determined using the 2^-ΔCt^ method versus GAPDH which was identified as the most appropriate housekeeping gene using the Normfinder algorithm and the standard deviation (SD) method. Gene expression data are presented as mean ± SEM, and samples compared for significance using ANOVA and unpaired *t* tests in Prism 6.0 (Graphpad).

### Optic nerve explant cultures

Optic nerve explant cultures were prepared from mice aged postnatal day (P)7–12, as described previously (Greenwood and Butt 2003). In brief, optic nerves were carefully dissected and maintained in pre-warmed (37 °C) and pre-gassed (95%0_2_/5% CO_2_) dissecting media, consisting of high glucose Dulbecco’s modified Eagle medium (DMEM) (Sigma-D5671) containing 10% foetal calf serum (Life Technologies), l-glutamine (Sigma) and 0.1% gentamycin (Life Technologies). From this point on optic nerves were kept under sterile conditions and cut into 1–2 mm fragments in filter sterilized pre-warmed dissecting media, using a scalpel blade. For further dissociation, optic nerve fragments were triturated and transferred onto poly-d-lysine/matrigel-coated coverslips. After 24 h, the dissecting medium was replaced with a low serum (0.5%) modified Bottenstein and Sato (B&S) culture medium (Bottenstein and Sato 1979), supplemented with 10 ng/ml recombinant human PDGF-AA (R&D Systems) and 0.1% gentamicin. After 3–4 days in vitro (DIV) the medium was replaced with maturation medium, B&S media supplemented with 0.5 mM dibutyryl cAMP, for up to 12 DIV, changing media every 3–5 days. Explant cultures were used for immunolabelling or calcium imaging after maturation at 8–12 DIV, equivalent to ≥ P20.

### Immunolabelling

Optic nerve tissue and explant cultures were fixed in 1% paraformaldehyde in phosphate buffered saline (PBS, pH 7.4); tissue and explant cultures were fixed for 1 h and 10 min, respectively, at room temperature (RT), followed by washes in PBS. For sectioning, optic nerves from P15 PLP1-DsRed or WT mice were placed in cryoprotectant (30% wv^− 1^ sucrose in PBS) overnight at 4 °C, then embedded in Cryo-M-Bed (Bright Instruments Company Ltd), before rapidly freezing at −80 °C until use. Longitudinal optic nerve sections (14 μm) were cut with a Leica CM3050 S cryostat at −21 °C and sections were transferred onto Polysine^®^ coated slides (Thermo-Scientific). After this, tissue sections and cultures were treated the same. Following washes in PBS for 30 min, a blocking stage was performed using 5% normal goat serum (NGS) in PBS for 1 h at RT; where primary antibodies targeted an intracellular epitope, Triton X-100 (Sigma) was included in the blocking solution (0.1% for tissue sections and 0.01% for cultured cells). Primary antibodies were diluted in blocking solution and tissues/cells incubated overnight at 4 °C; anti-STIM1, anti-STIM2, anti-ORAI1, anti-TRPM3 were raised in rabbits (Alomone) and used at 1:300; chicken anti-GFAP (Chemicon) was used at 1:500. Samples were then washed 3 times in PBS and incubated with the appropriate secondary antibodies conjugated with Alexafluor 488 or 568 (1:400, Life Technologies), DyLight™ 649 (1:200, Stratech) or TRITC (1:100, Sigma); counterstaining with Hoechst Blue (1:1000, Fisher) was used to label cell nuclei. Controls were carried out in which sections/cells were preabsorbed with antigen peptide overnight prior to incubation in the primary antibody. Following immunolabelling, coverslips/sections were mounted with Fluoromount-G^®^ (Southern Biotech). Immunohistochemical labelling was determined by confocal microscopy, based on 2–3 sections for each antibody from *n* = 3 optic nerves, or from *n* = 3 coverslips for explant cultures.

### Image capture and analysis

Immunofluorescence was detected using excitation wavelengths of 488 nm (green), 568 nm (red), 633 nm (far red) and 405 nm (blue), with an argon, HeNe1 and diode laser, respectively. Confocal images were captured using the 20× or 40× oil immersion objectives, and images acquired using multi-track sequential capture, with optimal detector gain and offset acquisition settings for pinhole diameter 0.13–0.3 airy units, with an average of four scans per image, to detect positive signal with minimal background and prevent cross-talk between channels. Identical settings were used to image negative controls. *z*-stacks were captured of 4–15 *z*-sections (voxel size 43–76 nm *x–y*, 76–283 nm *z*). Image analysis was carried out using Volocity 6.1 software (PerkinElmer). For confocal photomicrographs, two-dimensional flattened images of the *z*-stacks are presented of representative data, from 2 to 3 sections for each antibody from *n* = 3 optic nerves, or from *n* = 3 coverslips for explant cultures. Colocalization images were obtained using Volocity 6.1 software, as described previously (Mondragao et al. 2016), by thresholding to separate the positive signal (positive immunolabelling) from background, using the negative controls, and calculating the thresholded Pearson’s correlation coefficient (PCC) to generate a colocalization channel representing in three-dimensions the voxels in which the two channels overlap with the same intensity.

### Calcium imaging

Optic nerves from P8-P13 PLP-DsRed and WT mice were isolated intact and placed in aCSF, comprising (in mM): NaCl 133, KCl 3, CaCl_2_ 2.24, NaH_2_PO_4_ 1.2, MgCl_2_ 1.0, d-glucose 10, HEPES 8.55, pH 7.3. For calcium imaging, nerves or cultures of optic nerve glia were incubated in *a*CSF containing 4 µM Fluo-4/AM for 60 min at RT, as described previously (Hamilton et al. 2008). Fluo-4 loaded samples were transferred to a perfusion chamber under a Zeiss LSM510 Pascal Axioskop 2 confocal microscope and imaged using a 20×/0.50 WPh2 Achroplan water immersion lens. Fluo-4 was excited with 488 nm argon laser and emitted light was collected at 510–580 nm. A series of optical *z*-sections were collected, typically 7–8 sections at 2–3 μm intervals, every 350–700 ms, and analysed using the Zeiss LSM Image Examiner software (Zeiss, Germany). Glial cell bodies were selected as regions of interest (ROI) and changes in fluorescence intensity above baseline (ΔF/F) were measured in arbitrary units (AU). Data were expressed as mean ± standard error of the mean (SEM), where ‘n’ represents the number of cells, and experiments were repeated a minimum of 3 times (*n* = 3 nerves or coverslips); significance was determined by unpaired *t* tests, using Prism 6.0 (Graphpad). The tissues were continuously perfused via a multitap system that allowed rapid turnover of solutions. Pharmacological agents were dissolved directly in aCSF: ATP (Sigma, 100 µM), which evokes raised cytosolic Ca^2+^ in astrocytes and oligodendrocytes mainly via P2Y receptors and is a reliable indicator of cell viability (James and Butt 2001); the potent sarco-endoplasmic reticulum Ca^2+^ ATPase (SERCA pump) blocker thapsigargin (Tocris, 2–10 µM), which has been shown to cause influx of calcium into the cytosol due to intracellular calcium store depletion in glial cells (Gudz et al. 2006; Simpson and Russell 1997); and 2APB (Tocris, 50 µM), which blocks a range of TRP channels, including TRPM3, together with TRPC1, TRPC3, and TRPM7, and has been shown to abolish Ca^2+^ influx due to SOCC in astrocytes (Mandal et al. 2008). Calcium-free *a*CSF (ZERO-Ca^2+^) was prepared by removal of CaCl_2_, addition of the Ca^2+^-chelator 1 mM EGTA, and increasing HEPES to 10 mM to maintain osmolarity. Unless otherwise stated, ATP was applied by perfusion over the nerve for 60 s at the beginning and end of experiments, to confirm integrity of the cells, and a recovery period of 10 min in *a*CSF was allowed between tests pulses.

## Results

### Astrocytes and oligodendrocytes express Orai/Stim

**Fig. 1:**
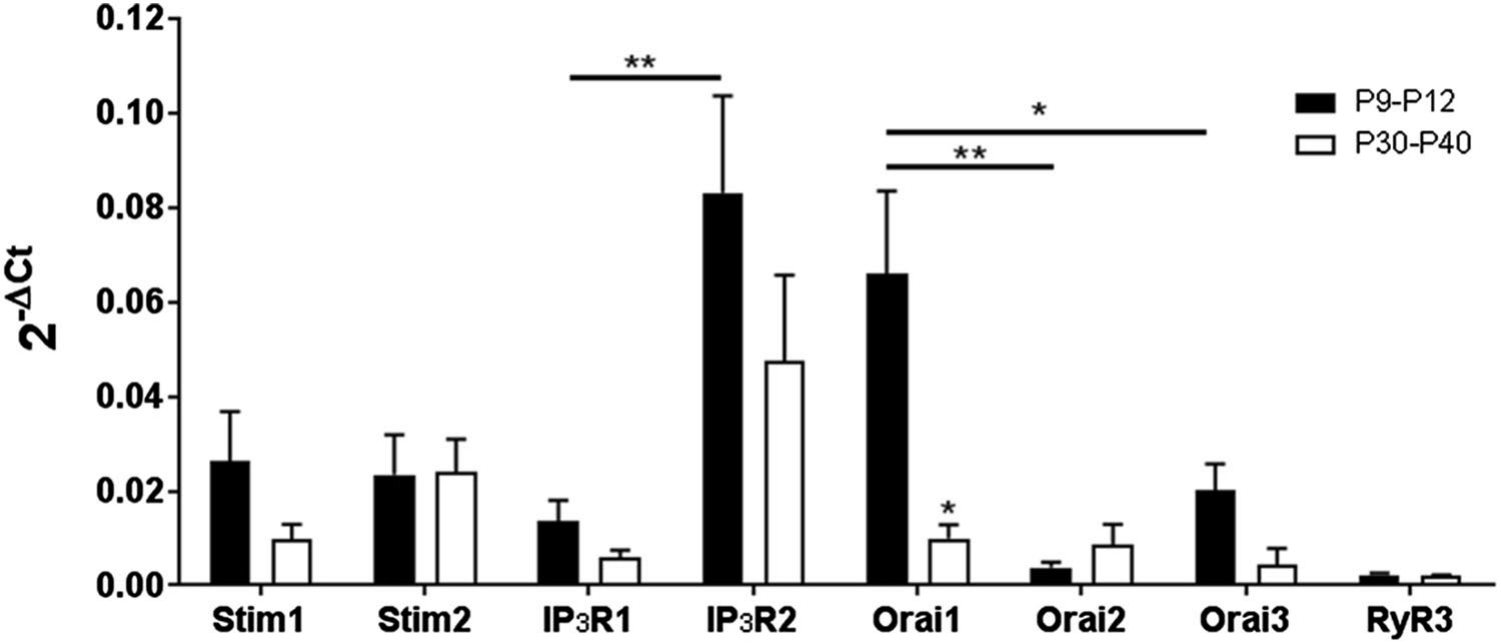
Expression of Orai/Stim transcripts in the mouse optic nerve. qRT-PCR was performed on lysates of acutely isolated optic nerves from WT postnatal mice (aged P9–P12) and adult mice (P30–P40). Data are from ten pooled optic nerves in each age group, run in triplicate, expressed as relative mRNA levels (2^-ΔCt^) compared to the housekeeping gene GAPDH (mean ± SEM, *n* = 3). Orai1 was the most highly expressed Orai isoform in the postnatal nerve (*p < 0.05, **p < 0.01, ANOVA and unpaired *t* tests) and was significantly downregulated with age (*p < 0.05, unpaired *t* test); there was no significant difference between the Orai isoforms in the adult. There was no significant difference between Stim1 and Stim2 expression in postnatal or adult nerves. IP3R2 was expressed at significantly greater levels than IP3R1 at both ages (**p < 0.01, unpaired *t* test); there was no significant difference between the age groups

To determine gene expression profiles of the main SOCE channels in postnatal and adult mice, we used qRT-PCR and normalized transcript levels against the housekeeping gene GAPDH by the comparative 2^-ΔCt^ method (Fig. 1); data are from ten pooled WT optic nerves in each age group, run in triplicate, with data expressed as mean ± SEM (*n* = 3). The results indicate that IP3 receptor 2 (IP3R2) is the primary ER Ca^2+^ release channel in optic nerve glia, with significantly lower expression of IP3R1 (*p* < 0.01, unpaired *t* test), whilst ryanodine receptor 3 (RyR3) was barely detectable; RyR3 is the main subtype expressed in the brain and RyR1 and RyR2 were not included in the Mouse Neuronal Ion Channels RT^2^ Profiler™ assay. Notably, all three Orai isoforms and both Stim1 and Stim2 were detected in the optic nerve (Fig. 1). Orai1 was the most highly expressed isoform in the postnatal optic nerve (*p* < 0.05, ANOVA) and levels of Orai1 were significantly lower in the adult nerve (*p* < 0.05, unpaired *t* test). There were no statistically significant differences in transcript levels of the three Orai isoforms in the adult nerve, or between Stim1 and Stim2 in postnatal or adult nerves (Fig. 1).

**Fig. 2:**
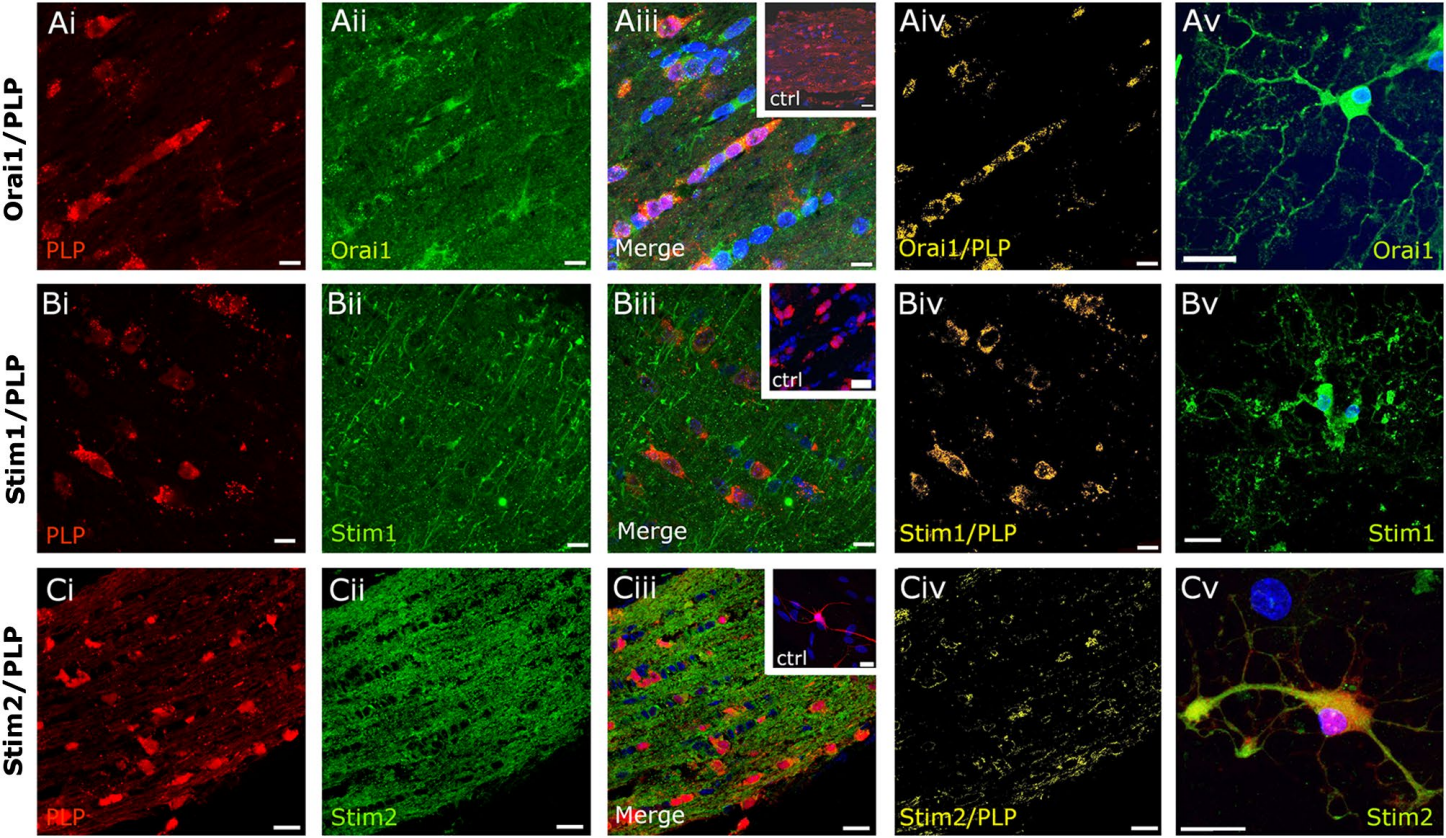
Expression of Orai/Stim in optic nerve oligodendrocytes. Immunolabelling for Orai1 (**a**, *green*), Stim1 (**b**, *green*) and Stim2 (**c**, *green*) in PLP-DsRed mice to identify oligodendrocytes (**a**–**c**, *red*), in optic nerve sections (**ai** *–* **iv, bi**–**iv, ci**–**iv**) and explant cultures (**av, bv, cv**). Confocal micrographs illustrate single channels (**ai, aii, bi, bii, ci, cii**) and merged cannels (**aiii, biii, ciii**). Expression of Orai1 and Stim1 is localized to oligodendroglial somata, whereas Stim2 immunostaining was primarily within the fascicles of myelinated axons, as demonstrated by the colocalization channels, illustrating voxels in which *green* and *red channels* are of equal intensity and appear *yellow* (**aiv, biv, civ**). Oligodendrocytes in explant cultures are immunopositive for Orai1 (**av**), Stim1 (**bv**) and Stim2 (**cv**). No immunostaining was observed in negative controls that were pre-incubated in blocking peptides for Orai1 (*inset*, **aiii**), Stim1 (*inset*, **biii**) and Stim2 (*inset*, **ciii**). Nuclei are stained with Hoechst blue. *Scale bars* **a, b** 10 µm, **c** 20 μm

**Fig. 3:**
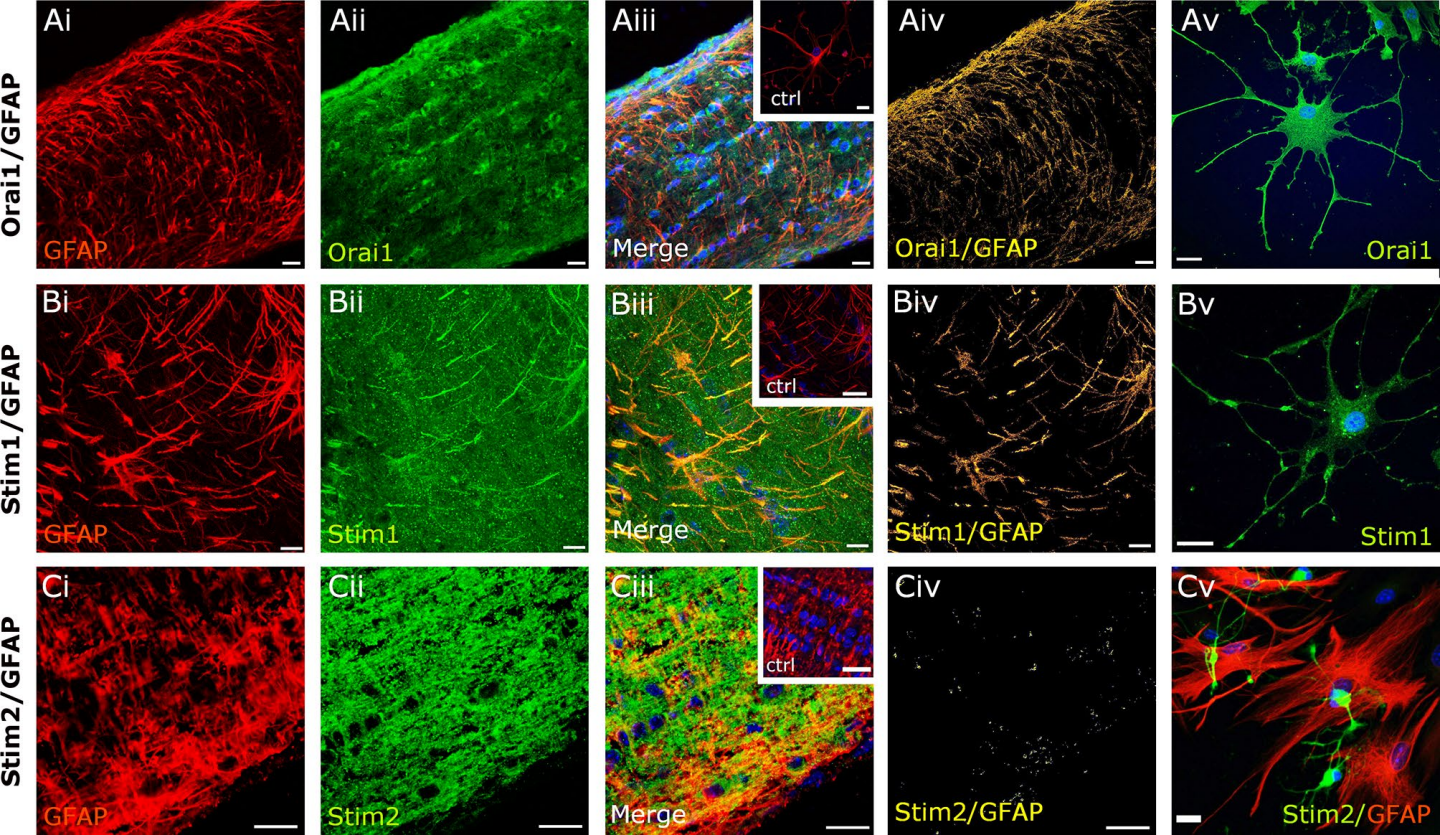
Expression of Orai/Stim in optic nerve astrocytes. Double immunofluorescence labelling for Orai1 (**a**, *green*), Stim1 (**b**, *green*) and Stim2 (**c**, *green*), with GFAP to identify astrocytes (**a**–**c**, *red*), in WT optic nerve sections (**ai**–**iv**, bi–**iv, ci**–**iv**) and explant cultures (**av, bv, cv**). Confocal micrographs illustrate single channels (**ai, aii, bi, bii, ci, cii**) and merged cannels (**aiii, biii, ciii**). Expression of Orai1 and Stim1 is localized to astrocyte processes, whereas astrocytes were immunonegative for Stim2, as demonstrated by the colocalization channels, illustrating voxels in which *green* and *red channels* are of equal intensity and appear *yellow* (**aiv, biv, civ**). Astrocytes in explant cultures are immunopositive for Orai1 (**av**) and Stim1 (**bv**), but are immunonegative for Stim2 (**cv**). No immunostaining was observed in negative controls that were pre-incubated in blocking peptides for Orai1 (*inset*, **aiii**), Stim1 (*inset*, **biii**) and Stim2 (*inset*, **ciii**). Nuclei are stained with Hoechst blue. *Scale bars* **a, b** 10 µm, **c** 20 μm

Immunolabelling was performed in optic nerve sections and explant cultures from PLP-DsRed mice to identify oligodendrocytes (Fig. 2) and from WT mice using GFAP immunolabelling to identify astrocytes (Fig. 3); in both mouse strains the pattern of immunostaining was the same for Orai (Figs. 2aii, 3aii), Stim1 (Figs. 2bii, 3bii) and Stim2 (Figs. 2cii, 3cii). No immunoreactivity was detected in negative controls following preabsorption with the antigen peptide for each of the primary antibodies used (insets in Fig. 2aiii, biii, ciii). The most prominent expression of Orai1 in optic nerve sections was in rows of oligodendroglial cell somata identified by expression of the PLP-DsRed reporter (Fig. 2ai–iii), which was confirmed by the generation of a colocalization channel that identifies the individual voxels in which the red (PLP-DsRed) and green (Orai1 immunostaining) channels overlap with the same intensity (Fig. 2aiv), as described previously (Hawkins and Butt 2013). The results demonstrate clear colocalization of Orai1 with PLP-DsRed in oligodendroglial somata in optic nerve sections (Fig. 2aiv), and the presence of Orai1 in oligodendrocytes is morphologically confirmed in optic nerve explant cultures (Fig. 2av). In addition, oligodendrocytes were immunopositive for Stim1 (Fig. 2bi–iii), which the colocalization channel indicates is strongest in oligodendroglial somata (Fig. 2biv). In contrast, Stim2 immunostaining was strongest within the myelinated axon fascicles between the rows of oligodendrocyte somata (Fig. 2 ci–iii), and is observed to decorate oligodendroglial processes in the colocalization channel (Fig. 2civ). Oligodendroglial expression of Stim1 and Stim2 was morphologically confirmed in cultured cells (Fig. 2bv, cv). Double immunofluorescence labelling of WT optic nerve sections with GFAP showed that astrocytes are immunopositive for Orai1 and Stim1 (Fig. 3ai–iii, bi–iii), but they were immunonegative for Stim2 (Fig. 3ci–iii), which was confirmed by generation of a colocalization channel in sections (Fig. 3aiv, biv, civ) and morphologically in cell cultures (Fig. 3av, bv, cv). The results support the potential importance of Orai1/Stim1 CRAC in white matter astrocytes and oligodendrocytes.

### TRPM3 are the main TRP channels in optic nerve astrocytes and oligodendrocytes

**Fig. 4:**
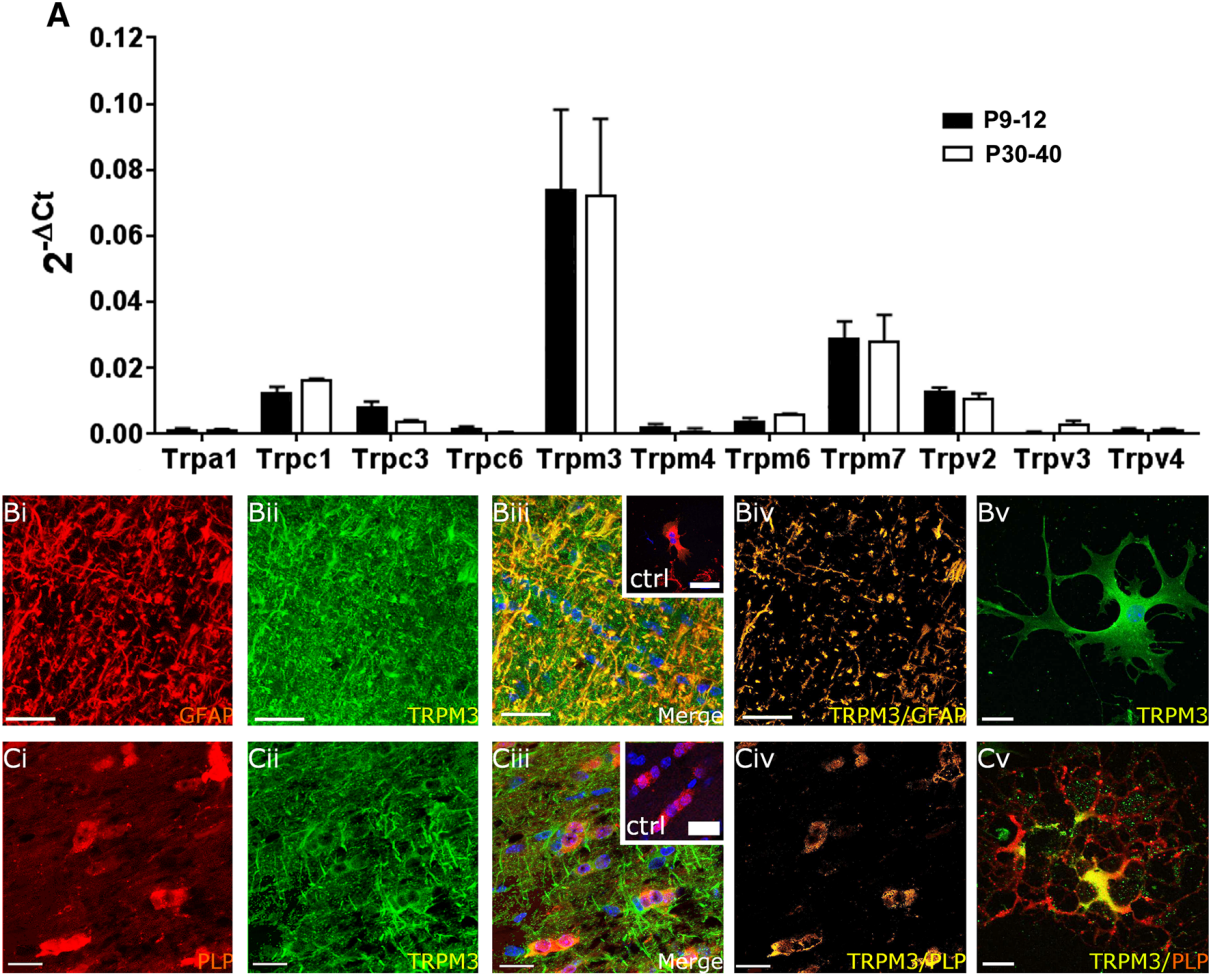
Expression of TRP channels in optic nerve glia. **a** qRT-PCR of acutely isolated optic nerves from WT mice aged P9–P12 and P30–P40; data are from 10 pooled optic nerves in each age group, run in triplicate, expressed as relative mRNA levels  (2^-ΔCt^) compared to the housekeeping gene GAPDH method (mean ± SEM, *n* = 3). TRPM3 was the most highly expressed TRP channel in the postnatal and adult nerve (***p < 0.001, ANOVA and unpaired *t* tests) and there was no developmental regulation of TRP channels, which had a rank order of expression in the adult of TRPM3 >>> TRPM7 (***p < 0.001, unpaired *t* test) > TRPC1 (*p < 0.05, unpaired *t* test) >> TRPV2 (**p < 0.01, unpaired *t* test); TRPC3, TRPM6 and TRPV3 were expressed at significantly lower levels, and TRPA1, TRPC6, TRPM4, and TRPV4 were barely detectable. Immunolabelling for TRPM3 (**b, c**, *green*) with GFAP to identify astrocytes in WT nerves (**b**, *red*) and in PLP-DsRed nerves to identify oligodendrocytes (**c**, *red*), in optic nerve sections (**bi**–**iv, ci**–**iv**) and explant cultures (**bv, cv**). Confocal micrographs illustrate single channels (**bi, bii, ci, cii**) and merged cannels (**biii, ciii**). The colocalization channels illustrate voxels in which *green* and *red channels* are of equal intensity and appear *yellow*, showing expression of TRPM3 is localized to astroglial processes (**bv**) and oligodendroglial somata (**cv**). Immunostaining of explant cultures shows astrocytes are immunopositive for TRPM3 (**b**v) and that TRPM3 is localized to oligodendroglial somata and excluded from their distal processes (**cv**). No immunostaining was observed in negative controls that were pre-incubated in blocking peptides for TRPM3 (*insets*, **biii, ciii**). Nuclei are stained with Hoechst blue. *Scale bars* 20 μm

qRT-PCR transcriptomic analysis indicated that by far the most abundant TRP channel in the WT postnatal and adult optic nerve is TRPM3 (Fig. 4a, p < 0.001, ANOVA and post hoc unpaired *t* tests), which mediates Ca^2+^ entry in response to a variety of different stimuli, such as sphingolipids and extracellular osmotic disturbances (Hoffmann et al. 2010). Immunolabelling for TRPM3 was performed on optic nerve sections and explant cultures from WT mice using GFAP immunolabelling to identify astrocytes (Fig. 4b) and PLP-DsRed mice to identify oligodendrocytes (Fig. 4c); the pattern of TRPM3 immunostaining was similar in both strains (Fig. 4bii, cii), and no immunoreactivity was detected in negative controls following preabsorption with the antigen peptide for the TRPM3 primary antibody (insets in Fig. 4biii, ciii). Double immunofluorescence labelling for GFAP and TRPM3 shows for the first time that astrocytes express TRPM3 in optic nerve sections (Fig. 4bi–iii) and astrocytic expression is morphologically confirmed in optic nerve explant cultures (Fig. 4bv). Colocalization analysis of TRPM3 and GFAP immunostaining indicated TRPM3 is localized mainly to astrocyte processes (Fig. 4biv). In addition, TRPM3 is expressed in PLP-DsRed + oligodendrocytes (Fig. 4ci–iii), in support of a previous study demonstrating TRPM3 in sphingosine-sensitive oligodendrocytes (Hoffmann et al. 2010). Generation of colocalization channels for PLP-DsRed and TRPM3 immunostaining indicates TRPM3 is highly localized to oligodendrocyte somata (Fig. 4civ), which is confirmed in cultured oligodendrocytes, where TRMP3 appeared to be excluded from distal oligodendroglial processes (Fig. 4cv). qRT-PCR indicated that other TRP channel subtypes included in the mouse neuronal array were expressed at much lower levels than TRPM3 (Fig. 4a). The second most expressed TRP channel transcript was TRPM7, followed by the ‘canonical’ TRPC1 and TRPV2, whereas TRPC3 and TRPM6 were expressed at low levels, and TRPA1, TRPV3, TRPV4, TRPM4 and TRPC6 were barely detectable, whilst TRPV1 and TRPM8 were not detected (Fig. 4a).

### Functional SOCE in astrocytes and oligodendrocytes

**Fig. 5:**
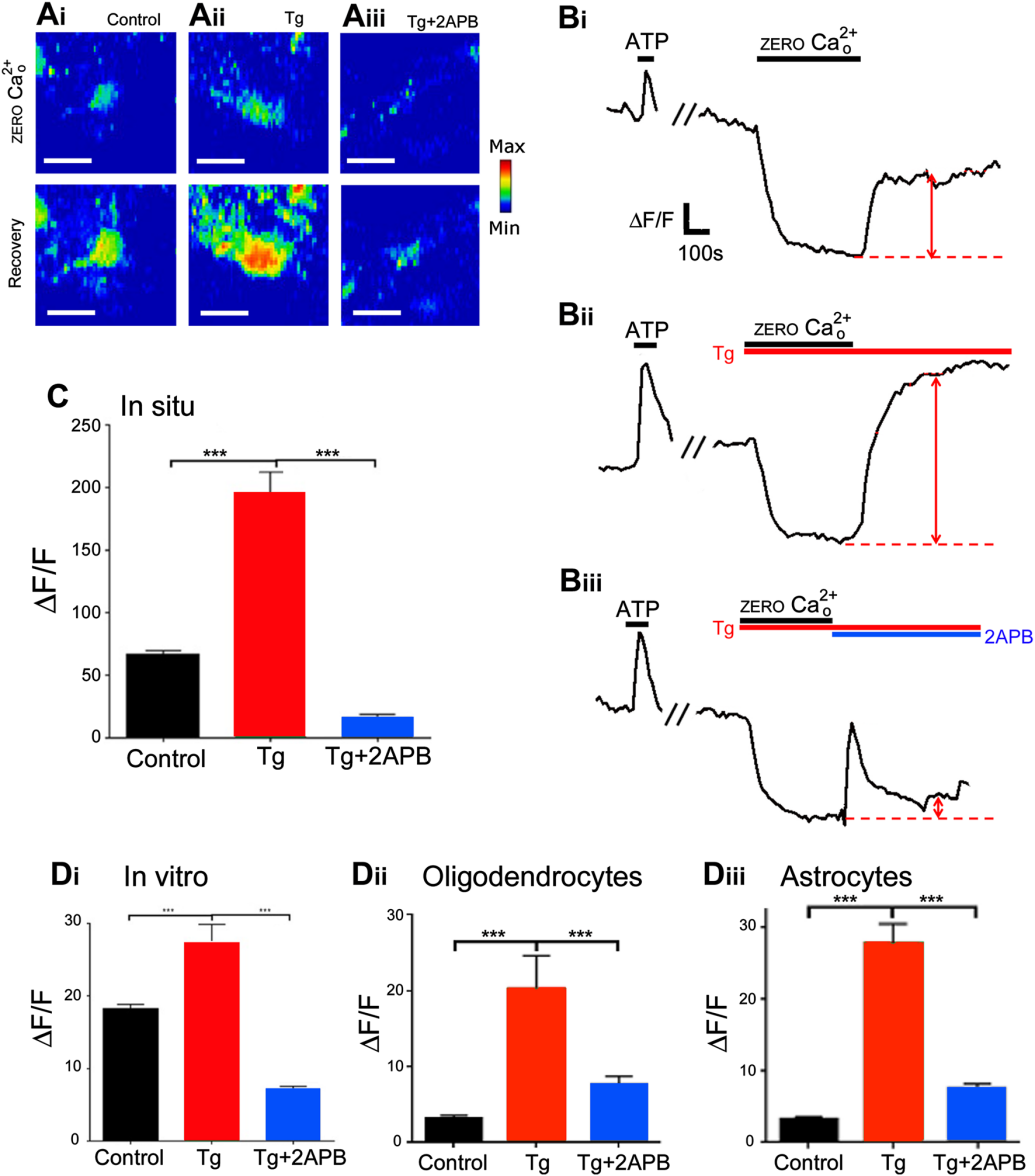
SOCE in optic nerve glia. Mouse optic nerves were isolated intact and loaded with Fluo-4 to analyse SOCE, using thapsigargin (10 µM for optic nerves, 2 μM for explant cultures) to block ER reuptake and 2APB (50 µM) to block SOCE channels. Confocal images of Fluo-4 fluorescence intensity illustrated in rainbow false colour (**a**, scale *bars* 10 μm) and representative traces of individual glia (**b**), illustrating that removal of extracellular [Ca^2+^]_o_ results in a decline in cytosolic [Ca^2+^]_i_, which recovers rapidly on return to normal *a*CSF (**ai, bi**, *red arrow*), and this is markedly increased in the presence of thapsigargin (**aii, bii**, *red arrow*) and decreased in the presence of 2APB (**aiii, biii**, *red arrow*). *Bar graphs* showing the mean rise in cytosolic [Ca^2+^]_i_ (indicated by *red arrows* in **bi**–iii) in *a*CSF control, thapsigargin and 2APB, in situ in the isolated intact optic nerve (**c**) and in vitro in explant cultures (**d**), illustrating results from all glia (**di**) and separately for oligodendrocytes (**dii**) and astrocytes (**diii**), identified by differential expression of PLP-DsRed; data are mean ± SEM change in fluorescence (ΔF/F), ***p < 0.01, unpaired *t* test with Welch’s correction

SOCE was examined by fluo-4 calcium imaging in isolated intact optic nerves (Fig. 5a–c), using the SERCA pump blocker thapsigargin (10 µM) to induce depletion of ER Ca^2+^ stores and stimulate SOCE (Moreno et al. 2012), and 2APB (50 μM), which is a potent blocker of SOCE channels at high concentrations (Abdullaev et al. 2008; Singaravelu et al. 2006). No difference was found between WT and PLP-DsRed strains and the data were combined. Removal of extracellular [Ca^2+^]_o_ resulted in a depletion of glial cytosolic [Ca^2+^]_i_, which recovered rapidly on return to normal [Ca^2+^]_o_ (Fig. 5ai), due to Ca^2+^ influx across the plasmalemma from the extracellular milieu, as illustrated by the representative images (Fig. 5ai) and traces (Fig. 5bi) of changes in glial cytosolic [Ca^2+^]_i_ in the isolated intact optic nerve. The recovery in glial cytosolic [Ca^2+^]_i_ was markedly greater and sustained in the presence of thapsigargin, which blocked SERCA-mediated removal of Ca^2+^ from the cytosol into the ER stores (Fig. 5aii, bii). Conversely, blockade of SOCE channels by a high concentration (50 μM) of 2APB completely abolished the recovery in glial cytosolic [Ca^2+^]_i_, indicating replenishment of intracellular Ca^2+^ stores is entirely dependent on Ca^2+^ influx from the extracellular milieu via SOCE (Fig. 5aiii, biii). Compared to controls (*n* = 81 cells from 5 nerves), the effects of thapsigargin (*n* = 37 cells from 3 nerves) and 2APB (*n* = 83 cells from 5 nerves) were statistically significant (Fig. 5c, p < 0.001, unpaired *t* tests with Welch’s correction). It is difficult to distinguish unequivocally between astrocytes and oligodendrocytes in the isolated intact optic nerve (Hamilton et al. 2008), hence we repeated the experiments on optic nerve explant cultures in oligodendrocytes and astrocytes identified by their differential expression of the oligodendroglial reporter PLP-DsRed (Brasko et al. 2017). The results demonstrate that the effects of thapsigargin (2 μM) and 2APB (50 μM) were equivalent in situ (Fig. 5c) and in vitro (Fig. 5di), and the same response was observed in both oligodendrocytes (Fig. 5dii) and astrocytes (Fig. 5Diii); in all cases, the effects of thapsigargin and 2APB in vitro were significant compared to controls (*p* < 0.001, unpaired *t* tests with Welch’s correction: controls, *n* = 91 cells from 3 coverslips; thapsigargin, *n* = 37 cells from 3 coverslips; 2APB, *n* = 198 cells from 5 coverslips). The results demonstrate functional SOCE in white matter astrocytes and oligodendrocytes.

**Fig. 6:**
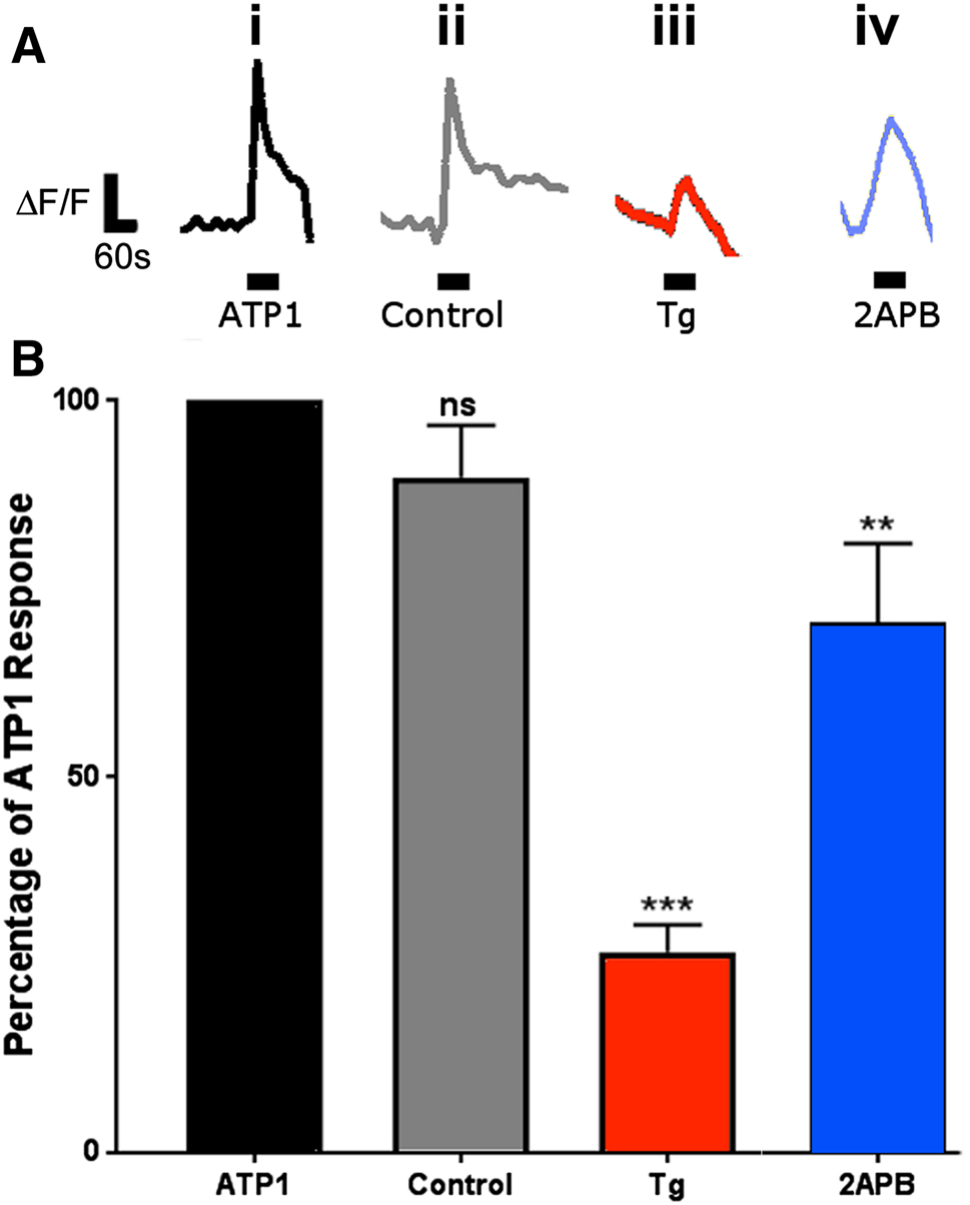
SOCE is essential for sustainability of ATP-mediated Ca^2+^ signalling in optic nerve glia. **a** Representative traces illustrating ATP-mediated rise in glial Ca^2+^ at the beginning of each experiment (**ai**, ATP1) and the second response tested at the end of the experiment, without application of any pharmacological agents (**aii**, control), compared to after application of thapsigargin to block replenishment of ER Ca^2+^ stores (**aiii**, Tg), or 2APB to block SOCE (**aiv**, 2APB). **b** *Bar graph* of mean (±SEM) responses to the second test pulse of ATP, expressed as a percentage of the first response to ATP at the beginning of the experiment (**p < 0.01, ***p < 0.001, unpaired *t* tests with Welch’s correction)

Notably, ATP-mediated calcium signals occur without decay following repeated applications of ATP and is the primary mechanism of calcium signalling in optic nerve glia (James and Butt 2001). This was reaffirmed by consecutive administration of two test pulses of ATP for 60 s, with a 5-min recovery period between pulses (Fig. 6ai, ii), and there was no significant difference in the amplitude of the rise in glial cytosolic [Ca^2+^]_i_ between first and second pulses in control *a*CSF (Fig. 6b, *p* > 0.05, unpaired *t* test with Welch’s correction; *n* = 50 cells from 3 nerves). In contrast, administration of thapsigargin to block replenishment of ER stores (Fig. 6aiii) or 2APB to block SOCE (Fig. 6aiv) resulted in a marked and statistically significantly attenuation in the response to the second application of ATP (Fig. 6b, *p* < 0.001 for thapsigargin (*n* = 37 cells from 3 nerves), *p* < 0.01 for 2APB (*n* = 104 cells from 6 nerves), unpaired *t* tests with Welch’s correction). The results demonstrate that SOCE-dependent replenishment of ER Ca^2+^ stores is essential for ATP-mediated calcium signalling in optic nerve glia.

## Discussion

**Fig. 7:**
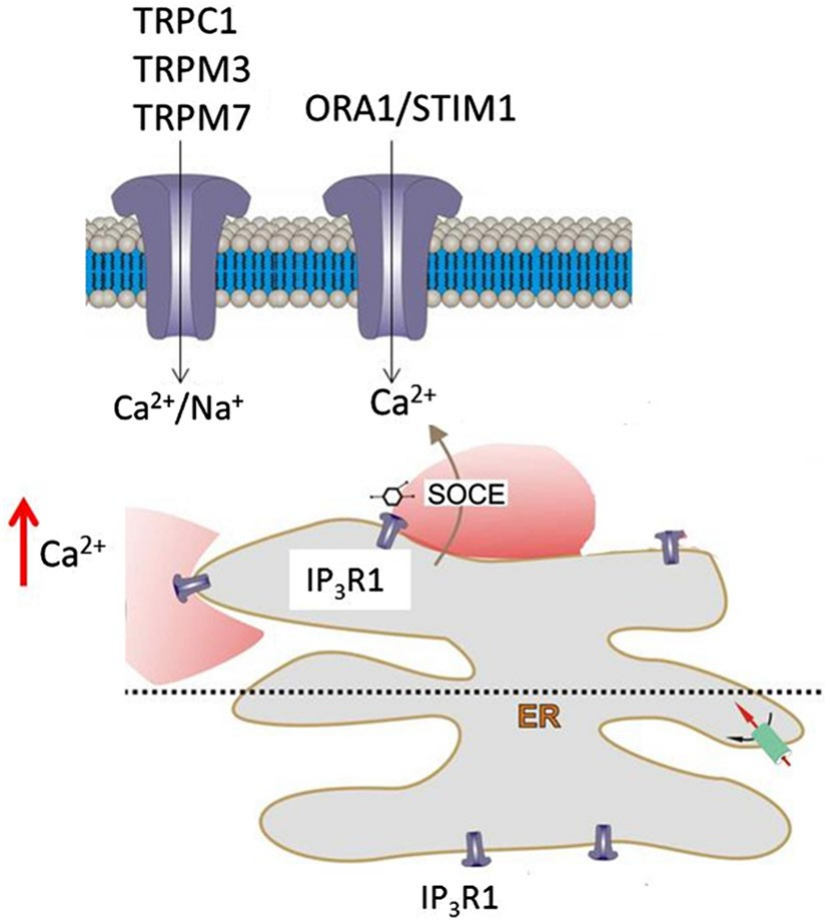
Mechanisms of SOCE in optic nerve glia. ATP-mediated calcium signalling in optic nerve glia is via P2Y G-protein-coupled receptors and the formation of IP3, which acts on IP3R1 on the ER to trigger release of Ca^2+^ into the cytosol. Subsequent replenishment of ER stores in astrocytes and oligodendrocytes is dependent on SOCE via TRPM3 and Orai1, which form the plasmalemmal Ca^2+^ channels, and mainly Stim1, which acts as the sensor of Ca^2+^ depletion, and uptake into the ER is via SERCA pumps. Oligodendrocytes also express Stim2, which may be localized to the myelin, whereas Orai1, Stim1 and TRPM3 are localized to oligodendroglial somata. Notably, calcium homeostasis in optic nerve glia depends on an apparent continuous Ca^2+^ influx from the extracellular milieu that is largely dependent on SOCE. Moreover, SOCE is essential for the sustainability of ATP-mediated Ca^2+^ signalling in optic nerve glia, which has a central role in white matter physiology and pathology

Calcium signalling is a ubiquitous physiological characteristic of glial cells, but the mechanisms of Ca^2+^ replenishment that sustain glial calcium signalling are poorly understood. Here, we demonstrate that white matter astrocytes and oligodendrocytes in the mouse optic nerve express Orai/Stim as well as TRPM3 channels, and the developmental downregulation of Orai1 is consistent with TRP channels being the dominant mechanism in mature astrocytes and oligodendrocytes (Verkhratsky and Parpura 2014). Furthermore, the results show that SOCE is essential for replenishment of intracellular ER Ca^2+^ stores and for the sustainability of ATP-mediated glial Ca^2+^ signalling, which is the primary mechanism of calcium excitability in optic nerve glia (James and Butt 2001). In white matter, ATP-mediated astroglial calcium signalling is triggered by axonal activity (Hamilton et al. 2008), acting to couple the homeostatic and metabolic support functions of glia to axonal activity and metabolic needs (Butt et al. 2014). The present study indicates Orai/Stim and TRPM3 are important elements in the calcium toolkit of optic nerve glia (Fig. 7).

SOCE plays important roles in the sustained phase of glial Ca^2+^ signalling observed following stimulation of G-protein-coupled receptors (GPCR) (Verkhratsky and Parpura 2014). Our results demonstrate SOCE is essential for ATP-mediated Ca^2+^ signalling in optic nerve glia, which has previously been shown to be primarily from IP3-mediated release of Ca^2+^ from intracellular ER stores (Hamilton et al. 2008; James and Butt 2001). As a typical CNS white matter tract, the optic nerve does not contain neuronal somata and the vast bulk of the transcriptome in whole optic nerve extracts represents mRNA from astrocytes and oligodendrocytes (>95%), with minor components from microglia and OPCs (together comprising ~5%), and trace levels from the vasculature and axons (Salter and Fern 2005). qRT-PCR of optic nerve transcripts identified high relative expression of IP3R2, which is consistent with it being the predominant IP3R involved in glial Ca^2+^ signalling (Fiacco and McCarthy 2006; Kanno and Nishizaki 2012). In addition, all three isoforms of Orai were detected by qRT-PCR, together with Stim1 and Stim2. Orai3 is the main microglial CRAC (Ohana et al. 2009), and is also expressed in OPCs (Paez et al. 2007). Our study identified Orai1 and Stim1 in astrocytes, but not Stim2, consistent with a previous report of Orai1/Stim1 forming the main CRAC in cortical astrocytes (Moreno et al. 2012). In contrast, oligodendrocytes were immunopositive for Orai1 and both Stim1 and Stim2, with a suggestion that Stim1 may be localized to the cell somata and Stim2 to the myelin. Interestingly, Golli, a member of the myelin basic protein (MBP) family of proteins has been shown to interact directly with Stim1 (Walsh et al. 2010), and to positively regulate myelin sheet expansion in cultured oligodendrocytes via mechanisms that require Ca^2+^ influx (Paez et al. 2007). In OPCs, the effects of Golli are mediated through the regulation of SOCE (Paez et al. 2009), which involves TRPC1 (Paez et al. 2011). Our findings provide a mechanism by which interactions between oligodendroglial Orai/Stim and Golli control Ca^2+^ influx and the regulation of myelination (Paez et al. 2007), possibly with a predominant role for Stim2 in Ca^2+^ handling specifically within internodal myelin sheaths. Calcium influx in myelin has been demonstrated in response to axonal electrical activity in the optic nerve (Micu et al. 2016), and is required for activity-dependent myelination (Howd et al. 1997) and oligodendroglial metabolic support for electrically active axons (Saab et al. 2016). Moreover, calcium handling is important for axon-myelin maintenance and integrity (Stys 2011), and Ca^2+^-mediated damage is a key feature of ischaemia and multiple sclerosis (Fern et al. 2014).

Interestingly, TRP channels are the main mechanism of SOCE in astrocytes and oligodendrocytes (Verkhratsky and Parpura 2014) and we detected an apparent developmental shift from equal levels of Orai1 and TRPM3 in the postnatal optic nerve to predominantly TRPM3 in the adult. This change corresponds to the major periods of myelination (Dangata et al. 1996) and maturation of astrocyte functions (Reemst et al. 2016), suggesting a potential greater role for Orai/Stim mechanisms of SOCE in immature glia and TRPM3 in mature glia. The most highly expressed TRP channel in the optic nerve was TRPM3, which we showed by immunolabelling is expressed at the protein level in both astrocytes and oligodendrocytes, supporting evidence that the most highly expressed transcripts in optic nerve reflect expression in astrocytes and oligodendrocytes (Salter and Fern 2005). TRPM3 has not previously been demonstrated in astrocytes, but has been identified in oligodendrocytes (Hoffmann et al. 2010), where it is highly localized to the cell somata and hence are most likely the primary molecular basis of SOCE in these cells. The rank order of expression of transcripts detected by qRT-PCR was TRPM3 >>> TRPM7 > TRPC1 > TRPV2 > TRPC3 ≥ TRPM6, with little evidence of developmental regulation between the postnatal and adult optic nerve. The low relative expression of transcripts other than TRPM3 may reflect they are of lesser importance in the predominant cell types, namely astrocytes and oligodendrocytes, and/or are potentially more highly expressed in the minor cell types, namely microglia and OPCs (Salter and Fern 2005). Supporting this, TRPM7 was the second most expressed transcript in the optic nerve and is one of the main TRP channels in microglia (Jiang et al. 2003) and RNA-Seq indicated TRPM7 is the most highly expressed TRP channel transcript in OPCs (Larson et al. 2016). Similarly, transcripts for TRPC1 and TRPC3 were very low in the optic nerve and are implicated, respectively, in OPCs (Paez et al. 2011) and microglia (Mizoguchi et al. 2014). In addition, the low levels of mRNA may reflect low levels of expression in astrocytes, since TRPC1 and TRPV2 are important in astrocytes (Golovina 2005; Parpura et al. 2011; Shibasaki et al. 2013), the latter also being implicated in oligodendrocytes (Fusco et al. 2004), whilst TRPM6 transcripts have been detected in astrocytes of the mouse optic nerve head (Choi et al. 2015). Notably, TRPM6 is central to Mg^2+^ homeostasis (Runnels 2011), and Mg^2+^ deficiency results in hypomyelination in the optic nerve (Gong et al. 2003), raising the possibility that TRPM6 may have an important Mg^2+^ regulatory function in myelination. Transcripts for TRPA1 and TRPV4 were barely detectable in our study, although TRPA1 is linked to astroglial GABA transport (Shigetomi et al. 2011), which is important in the optic nerve (Butt et al. 2014; Howd et al. 1997), and TRPA1 have been implicated in myelin damage in ischaemia (Hamilton et al. 2016), whilst TRPV4 are implicated in ischaemia in astrocytes (Butenko et al. 2012).

In summary, the results demonstrate SOCE is essential for calcium signalling in optic nerve glia, which has a central role in white matter physiology and pathology, including ischaemia, traumatic injury and multiple sclerosis (Rivera et al. 2016; Butt 2011; Fern et al. 2014). A striking observation is the dependence of glial Ca^2+^ homeostasis on an apparent continuous Ca^2+^ influx from the extracellular milieu, since removal of [Ca^2+^]_o_ resulted in a rapid and marked depletion of [Ca^2+^]_i_ within minutes. The effects of thapsigargin and 2APB, which at high concentrations is a potent inhibitor of Orai and TRP channels (Peinelt et al. 2008; Prakriya and Lewis 2001; Trebak et al. 2002), indicate this is largely dependent on SOCE (Fig. 7). Based on our expression data, the main mechanisms of SOCE are likely to involve TRPM3 and Orai1, which form the plasmalemmal Ca^2+^ channels, and mainly Stim1, which acts as the sensor of Ca^2+^ depletion (Cahalan 2009; Huang et al. 2006; Soboloff et al. 2012). Moreover, TRP channels are permeable to both Ca^2+^ and Na^+^, providing a potential link between glial Ca^2+^ and Na^+^ signalling, which together interact to mediate neuronal–glial interactions (Rose and Verkhratsky 2016).
